# Cases of Erysipelas Accompanied by Affection of the Throat; with Remarks on the Propriety of Limiting the Application of the Term

**Published:** 1827-03

**Authors:** James M. Arnott

**Affiliations:** Surgeon.


THE LONDON
### Medical and Physical Journal.

Nº 337, VOL. LVII.] MARCH, 1827. [Nº 9, *New Series.*

For many fortunate discoveries in medicine, and for the detection of numerous errors, the world is indebted to the rapid circulation of Monthly Journals; and there never existed any work, to which the Faculty, in Europe and America, were under deeper obligations, than to the Medical and Physical Journal of London, now forming a long, but an invaluable, series.—RUSH.

### ORIGINAL PAPERS,
AND
### CASES OBTAINED FROM PUBLIC INSTITUTIONS AND OTHER AUTHENTIC SOURCES.

### ERYSIPELAS.

*Cases of Erysipelas accompanied by Affection of the Throat; with Remarks on the Propriety of limiting the Application of the Term.* By JAMES M. ARNOTT, Esq. Surgeon.

THE connexion of erysipelas of the face with an inflammatory affection of the throat, is a circumstance to which attention has not hitherto been much directed; whilst the question of the contagious nature of the disease is one still considered *sub judice*. Were the following cases solitary examples of the former pathological fact, they would not perhaps merit being recorded; but, taken in conjunction with others which have been recently published,* they may probably possess some interest; and they bear also upon the question of the occasionally contagious character of the disease. In the remarks which succeed, I shall endeavour to employ these cases for the elucidation of the nature of erysipelas of the face itself, and afterwards attempt to show the propriety of restricting the application of this term, to one morbid condition, instead of continuing to apply it, as at present, to several having little pathological relationship.

The cases occurred in one family. The mother was first affected with inflammation of the pharynx, terminating in mortification. On her death, the husband was attacked with inflammation of the throat and erysipelas of the face. As he

* By Dr. STEVENSON, in the second volume of the Transactions of the Medico-Chirurgical Society, Edinburgh, 1826.

recovered, the daughter was similarly seized with inflammation of the pharynx and severe erysipelas.

Case I.—June 22d, 1826, Mrs. M—, ætatis forty-five, of spare make and delicate constitution, complained of pain and difficulty of swallowing. On examination, no redness could be perceived in the posterior fauces, and externally there was neither tenderness nor swelling. Tongue clean; no headache nor febrile disturbance. Attributed the attack to exposure to cold, whilst overheated, on the evening of the 20th instant.

She was ordered an emetic, a dose of Calomel and James's Powder, and subsequently a saline aperient.

23d.—Symptoms of affection of the throat as yesterday; but, on re-inspection, no redness could be discovered either of the velum, tonsils, or posterior pharynx, and the tip of the epiglottis was likewise seen to be of its natural colour. She refers the pain, which is only felt on attempting to swallow, to a situation behind the larynx, but pressure here does not give pain. Slight heat of skin, with some frequency of pulse; tongue clean and moist.

Leeches to the throat externally; saline medicine with Tartarised Antimony.

24th.—Sent for early to this patient, who had a restless night, owing to a distressing sensation of dryness and constriction in her throat, whenever she dropt off to sleep. Pain and difficulty of swallowing increased, and she complains of a troublesome quantity of phlegm in her throat, the excretion of which is attended with much pain. Some tumefaction in the anterior part of the neck, in the region of the thyroid gland, more particularly of its right lobe; and the skin is here tense and tender to the touch. Tongue moist, but has a patchy appearance; portions of its surface being white, whilst the rest is of its natural red colour. Slight hoarseness; no cough; respiration free; pulse frequent, of moderate volume, but resisting.

Eighteen ounces of blood were taken from the arm, and leeches were ordered to the throat externally.

Four hours afterwards, the blood drawn was cupped and buffy; the tension of the front of the neck had abated; she expressed herself much relieved; swallowed more easily, and had got down a dose of aperient mixture. She continued apparently better until towards night, when she became restless, with a slight flush on each cheek, and a countenance for the first time expressive of anxiety. Respiration free; pulse frequent, of rather diminished volume, but without resistance. I had this evening the benefit of Dr. Macleod's assistance, and it was resolved to give an opiate.

Symptoms of sinking, (apparently dependant on mortification having taken place,) continued to manifest themselves, and, early on the morning of the 25th, stimulants and tonics were resorted to. She rallied a little towards afternoon, but got worse at night, and died early on the 26th.

On dissection, the inflammation was found to have been of very limited extent, occupying that portion of the pharynx only which immediately borders on the aperture of the larynx. The limits of the inflamed part might be included within those of a shilling; on one edge of this portion (that forming part of the laryngeal aperture,) was a small spot of mortification, not much larger than that of a silver penny. The slough (for it was surrounded by a distinct line, as if nature had begun the work of separation,) occupied the right lip of the opening of the larynx, from the root of the epiglottis to the corresponding arytenoid cartilage, without, however, descending into the cavity of the larynx itself, which was free from disease. The vessels of the thyroid gland were more loaded than natural.

Case II.—June 29th, Mr. M—, three days after the death of his wife, with whom he had been in constant communication, complained of having been unwell for the last two days. Yesterday his throat felt sore; to-day it has been worse, with painful deglutition, and he expressed apprehensions that he was going to be attacked with the same disease as his wife. On examination, there was found a diffused redness all over the posterior fauces, but without much swelling. Next day (30th) the inflammation of the velum, tonsils, and posterior pharynx had increased, and a blush of redness, with tumefaction of the right upper eyelid, was observed. On the 1st of July, erysipelas had developed itself in the eyelid, and was extending on the cheek.

It is unnecessary to give the progress of the case in detail: the attack proved severe, the erysipelas having occupied the face, extended itself to the skin of the scalp, and did not complete its circuit of the head till the 11th or 12th; and it was not until the 22d that Mr. M— was able to sit up. Abscess formed in the eyelid primarily affected. The affection of the throat continued for a few days, and then disappeared. About the fifth or sixth day of the disease, the bowels became extremely irritable, and numerous motions were passed,—at first of offensive feculent matter, afterwards of thin, glairy, yellowish mucus. This last condition of the evacuations continued for several days.

The treatment pursued was in the first instance strictly antiphlogistic: when the violence of the disease had abated, stimulants and tonics were resorted to, with opiates at night to procure sleep. It may, however, be mentioned that some wine, given at a time when the patient was apparently much reduced, and was thought by his friends to be sinking from the effects of the purging, evidently did harm, and was not repeated.

Case III.—Miss M—, ætatis twenty, lived in the country at the time of her mother's death, and did not arrive in town until the day following. She frequently nursed her father during his illness, and at a time when the diseased skin in him was in a state of desquamation, first complained of being unwell,—viz. of headache and sore-throat, on the evening of the 22d July. On the 23d, I found her, with considerable febrile disturbance, complaining of great pain and difficulty in swallowing; and, on inspection, the velum palati, uvula, and tonsils were found of a bright red colour, with some swelling of the parts. The posterior surface of the pharynx was covered by a layer of yellow glairy mucus, not unlike the coagulable lymph found in the abdomen of women who have died of peritoneal inflammation after delivery. It could not be detached from the surface which it covered, but at one point where this was seen, there did not appear to be any abrasion. The patient complained of stuffing of the nostrils, and the left ala nasi was observed to be slightly tumid. On the 24th, vesicles appeared on the nose, and erysipelatous redness occupied the left cheek. After this the disease developed itself successively in the face, ears, the hairy scalp, first on the one side and then on the other, completing the circuit of the head by the 2d of August.

In this case the attack was more severe than in the preceding: abscesses formed in three of the eyelids; the vascular disturbance was so considerable as to require the abstraction of blood; and the affection of the bowels was likewise greater in degree. This state of bowels began on the fifth day of the disease, and continued for eight or nine days; the motions passed were numerous, and consisted of a thin, watery, yellow mucus, like yolk of egg beat up in water. When not checked by medicine, the number of motions and quantity of evacuation was so considerable as to enfeeble the patient. At first a combination of Hydrarg. cum Creta and Opium, and afterwards of Chalk Mixture with Black-drop, were employed to moderate the irritation of the bowels. The dose given at bed-time was intentionally omitted one night, and the consequence was that seventeen motions were passed in the course of it, inducing an unpleasant appearance of sinking; but out of which the patient rallied by simply checking the action of the bowels. The inflammatory affection of the throat continued several days, and then ceased.

The antiphlogistic treatment was adopted at the commencement of this case, as in the preceding; with the addition of a single venesection, to the extent of a pint. At a subsequent stage tonics were resorted to, but no wine.

The first of these cases presents rather an unusual instance of the small extent of disease which is occasionally sufficient to produce death. The symptoms of local affection were never such as to cause apprehension, nor the constitutional such as to excite alarm for the event, until those of sinking appeared. Indeed, we do not readily perceive why so small a spot of mortification should have been attended with fatal consequences; for that it was the occurrence of this, and not the very limited extent of inflammation, which produced death, seemed very evident. Had the situation of the gangrenous spot any connexion with the fatal issue? It occupied the lip of the laryngeal aperture; the mechanical part of respiration was not, however, affected. Was there any thing mysterious in the kind of inflammation? The case did not give that impression at the time of its occurrence. The patient was of a feeble constitution naturally, rendered still more so by suckling.

With regard to the other two cases (of well-marked idiopathic erysipelas), it seems to me that their origin was entirely owing to the existence of the preceding case. The father derived his disease from his wife; the daughter (who had not seen her mother) from her father. Had the first related case not have existed, the other two would never have occurred. In stating this, it is simply meant to be asserted that the latter owed their origin to a morbid profluvium derived from a body labouring under disease. The affection of the throat, in both cases, preceded that of the integuments of the face. Another circumstance worthy of remark, was the peculiar affection of the bowels, which occurred during the existence of the inflammation in the face, and supervened upon that of the fauces. This irritable state of bowels I have likewise seen in another case of erysipelas with affection of the throat, which I shall afterwards have occasion to notice. The time of its appearance, its course, and the character of the evacuations, leave little doubt that this affection of the intestinal canal was dependent on a state of mucous membrane similar to that which was observed in the pharynx. If such inflamed condition of the mucous membrane of the stomach and bowels exists in erysipelas, is this not sufficient to account for the state of the tongue, and other supposed bilious characters of the disease, without its being necessary to refer to an affection of the liver, the proofs of which are null?

The first, and indeed it may be said only, writer who has directed attention expressly to the connexion of erysipelas with an inflammatory affection of the throat, is Dr. STEVENSON, in the paper already referred to. In stating this, I am not unaware that such an affection has been casually alluded to by some of the writers on Erysipelas, as an occasional occurrence from the extension of the inflammation from the face to the fauces, but they have done so in an incidental manner, and without appearing to attach any importance to it. Dr. S., however, has described it in a more precise manner, and not as a consequence but as a precedent to the affection of the integuments; as one, also, which may occur in persons who have been much with erysipelatous patients, without the supervention of disease of the skin. To illustrate the connexion between the two affections, twenty-one cases are selected and given, although many more occurred in the author's practice. Of these, the origin of two (Cases 1st and 11th,) is not accounted for, and we may for the present consider them as having originated spontaneously: the remaining nineteen were all traced to other cases already existing. In analysing these last, for an object which will subsequently appear, it will be found that they stand thus—

Five of erysipelas simply (four of the face and one of the arm).

Three of erysipelas of the face and affection of the throat conjoined.

Ten of affection of the throat simply.

One of affection of the throat, with erysipelas supervening on the chest after the application of a blistering plaster to the neck.

The affection of the throat was characterised by a red or purplish blush of the velum pendulum and uvula, with very little tumefaction, but considerable pain in swallowing; excoriation of the inflamed surface frequently occurred, and superficial ulceration. It was ushered in by febrile symptoms, generally severe, even in the milder cases; and the period at which it appeared after the accession of the fever varied from the second to the sixth day. In all the cases of erysipelas having affection of the throat in conjunction with it, the latter preceded the affection of the face; in no case was the reverse observed. The inflammation of the throat occurred also so frequently in persons who had been much with patients labouring under erysipelas, that Dr. S. could not doubt their identity, and he came to the conclusion that it was erysipelas of the fauces. In either case the most successful treatment was found to be copious blood-letting, and the other parts of the antiphlogistic treatment.

Although the above-mentioned writer be the only one who has called our attention expressly to this subject, evidence of the connexion of erysipelas with an inflammatory affection of the throat are to be met with in the observations of other writers, whose object has not been so specially to notice it.

It is well known that the late Mr. Newby died in consequence of a puncture received in opening the body of a child, which died of enteritis, having also, it is said, erysipelas of the abdomen. What the disease was of which Mr. N. died, it is not our object at present to inquire: the following circumstances, however, which took place in his family are of some interest to our present inquiry. The account is given by Dr. NELSON, after his detail of the case.* "It is worthy of remark that, during Mr. Newby's illness, Mr. Jackson, his assistant, had *an inflammation of the fauces, of an erysipelatous appearance,* which terminated in suppuration of the tonsil. His pupil had an attack of low fever, which continued about a week. The house-maid was severely affected with *cynanche tonsillaris,* which terminated by resolution. The nurse had a slight attack of pyrexia, with *pain and stiffness of the neck,* on account of which she went home for a day or two; but, returning to the house, she was attacked with *erysipelas phlegmonodes,* which proved fatal. Another woman, who assisted in the room, had also the *erysipelas phlegmonodes,* but recovered."

Referring to some of the more modern medical periodical publications for cases of idiopathic erysipelas given in detail, in order to ascertain what evidence they might afford of the existence of affection of the throat in this disease, the contrast presented by the histories of *idiopathic* erysipelas, and of what is called *traumatic* erysipelas—viz. inflammation of the skin, cellular substance, or fasciæ, from local injury, is rather striking. Of the first affection the cases are very few, and the notices brief: of the latter the number is considerable, and the details given at length. Indeed, the only exception to this remark, and the only cases adapted to our inquiry, I have met with (although it must be owned the researches have not been very extensive,) are some related by Dr. DUNCAN, jun.† for the purpose of showing the utility of venesection in this disease. But, of the ten cases he has detailed at length, not one half are cases of idiopathic erysipelas, six being cases of inflammation of the skin, originating from some local cause of irritation, in patients in the hospital for other complaints. Thus in Case

2d, it arose from injury of the head;

3d and 4th, it attacked the mammæ after the application of blistering plasters to the chest;

6th, it came on after a blister applied behind the ear;

7th, it attacked the lower extremities of a patient admitted with œdematous affection of the leg and great thickening of the skin, resembling elephantiasis.

9th, it attacked the face of an individual having a disease of the throat resembling sibbens, on the application of a leech to a swelling of the ala nasi.

The remaining four cases are the only ones of idiopathic

* Medical and Physical Journal for August 1823, p. 177.

† Edinburgh Med. and Surg. Journal for October 1821, p. 537.

erysipelas, and that too of the face. In them we find the following evidence of affection of the throat:—In Case 1st, in the report on the second day after admission, it is stated that she complained "*of severe sore-throat,*" but the appearances are unfortunately not given. Case 5th was admitted on the 13th of May, with "*cynanche tonsillaris,*" the erysipelas appearing on the 17th. Of Case 10th it is said, on the day of admission, "Has a slight cough, with increased secretion of saliva, and some viscid expectoration. *The velum and uvula are also covered with small white specks.*" In the 8th Case alone no allusion is made to disease of the fauces: so that, out of the four, there are three having evidence of such affection.

A good illustration of the connexion of idiopathic erysipelas of the face with affection of the throat occurred not long since, in a patient in St. Bartholomew's Hospital, under the care of Mr. Lawrence. The phenomena and progress (which I witnessed) were very similar to those I have above detailed, only not so severe: there was the same affection of the throat, of the bowels, and appearance of the evacuations passed. The case has been reported in the Lancet of December 9th, p. 336, under the title of "Pharyngitis with Erysipelas."

These observations are sufficient to show that an affection of the throat is not of rare occurrence in this disease: but, in perusing them, it will probably also have been remarked, that it is only in cases of idiopathic erysipelas of the face that this affection has been observed; a circumstance which might give rise to the suspicion that there may be something peculiar in the nature of idiopathic erysipelas of the face. This suspicion will be increased on finding that a certain coincidence as to situation prevails in those instances where the disease has originated from contagion, the face being in such cases also the seat of the affection of the skin. It seems almost unnecessary to mention that instances of inflammation attacking the skin in the vicinity of wounds or ulcers, are not included in this statement: these are cases of disease connected with local injury; whereas it is of idiopathic erysipelas alone we now treat. In the two cases, then, which have been detailed by myself, the face was the seat of disease;—in seven out of eight cases given by Dr. Stevenson as originating from contagion, the erysipelas was of the face;—and, although Dr. Nelson has not distinctly specified the seat of the disease in the cases which occurred in Mr. Newby's family, there is presumptive evidence that the situation was here likewise the same. The exception in Dr.

Stevenson's eighth case, where it affected the arm, I am not disposed to attach much importance to: we are well aware how trivial a cause of local irritation will, under certain circumstances, originate inflammation of the skin; and, had the details of Dr. S.'s cases been given at greater length, (they are included in three or four lines,) some cause might have been discovered to account for this solitary instance. But it may be objected by some, that, in considering the above cases as arising from contagion, I am assuming that which is not yet proved. To this I can only reply, that the subject does not seem capable of demonstrative proof; and that, in the absence of this, the evidence offered by the history of these cases appears to argue an origin from contagion as at least extremely probable. The supposition of a morbid profluvium derived from a body labouring under disease, accounts much more satisfactorily to my mind for the origin of these cases, than any other cause or combination of causes.

Let us refer also to cases of erysipelas reported by other writers as arising from contagion, and where no affection of the throat prevailed or is noted, and we shall find a similar coincidence as to situation. Dr. Wells* has recorded a number of instances to show the contagious character of this disease; some furnished by his own observation, others contributed by Dr. Pitcairn, Mr. Whitfield, and Dr. Baillie. Excluding the primary cases (amongst which, the cases of the lady in childbed and her infant, p. 220, must be ranked, as it does not appear which was first affected,) those originating from contagion amount to sixteen. Of the sixteen, fifteen are cases of erysipelas of the face. The only exception, that of Mrs. Emerton, does not appear to have been a case of erysipelas, as the following extract will show. Dr. Wells found her labouring under the ordinary symptoms of what is called low fever. "There were besides, upon several parts of her skin, irregularly shaped patches of a bright red colour, and of the size nearly of a half-crown piece; but the parts so affected were not elevated, and gave no pain upon being touched. One of her arms, however, was considerably swelled, and appeared livid; but there was no visible disease of the outer surface of the true skin of the arm, nor was the scarf skin separated from it. She died in the course of the same day." In Dr. Baillie's communication it is stated that, "during a part of the years 1795 and 1796, erysipelas of the face was much more

* Transactions of a Society for the Improvement of Medical and Chirurgical Knowledge, vol. ii. p. 213.

frequent in St. George's Hospital than he had ever before known it to be. Many persons were attacked by this disease after they came into the hospital; and, as the number of cases of it in a particular ward was much greater than in any other, he was hence led to suspect it was contagious, &c."

Dr. Dickson, formerly of Clifton, and lately of the Naval Hospital, Plymouth, has given* some cases in proof of erysipelas being occasionally propagated by contagion. The first was in the wife of a gentleman, who, after being exposed to great fatigue, wet, and cold, in extinguishing a fire that had broken out upon his premises, had an attack of erysipelas of the face, with considerable fever and delirium. As it began to decline, his wife, who had nursed him, and occasionally lain upon his bed, was attacked with the same disease precisely, with a great degree of fever and very violent delirium. Another instance occurred under the following circumstances:—The gardener of a gentleman, occupying a small house in the garden, was seized with erysipelas of the face: all intercourse between him and the rest of the family was cut off, and the butler alone was directed to carry him whatever he wanted. Exactly as in the preceding instance, as soon as the gardener began to recover, the butler was attacked with erysipelas.

Along with these two is given a third instance, where the disease was in the arm; but this was certainly not erysipelas, although it was attended with inflammation of the skin. A young lady, in consequence of a blow, abraded the skin near the elbow; inflammation took place; "leeches were applied to some part of the arm, which in many places presented hard knobs;" "abscesses continued to form in the course of the principal lymphatics, from the elbow to the axilla." This young lady's mother slept with her, and, after the abscesses in her daughter's arm had been opened and were discharging, she complained of pain at the point of the middle finger of her right hand: "she does not *believe* the cuticle was abraded. On the next day, *the inflammation extended upwards to the bend of the arm, in a narrow line, not exceeding in breadth the sixth or fourth part of an inch,*" —sufficient, without going further into the case, to show that the disease was not erysipelas. I ought to state, in justice to Dr. Dickson, that it seems doubtful if the last case was furnished by him.

In the Medical and Physical Journal for April last, Mr. Blackett has related four cases of what are entitled

* In the Medico-Chirurgical Journal for April 1819, p. 615.

Erysipelas. But, of these four, three are cases of inflammation of the extremities, arising from injury: one from abrasion of the skin of the ankle, another from wound of the hand by a fork, and a third from wound of the foot by a nail. The seat of the inflammation in these three cases we need not at present inquire into. The fourth case is the only one in which idiopathic erysipelas occurred, and *that of the face,* in a young lady. The attack seems to have been severe; and, after having detailed the particulars of it, Mr. B. observes, ' I must add, that, during this young lady's sickness, the nurse and servant in attendance were both attacked with erysipelas;" and, in some other remarks upon the same case, we find it stated, " It (erysipelas) was contagious in one family that came under my observation. Mr. ——, his lady, the nurse, and two servants, died."—Had the disease its seat in the face in these last cases also?

From the preceding observations, then, it would appear that erysipelas of the face has been repeatedly observed to be derived from contagion; and that it is frequently found to have an affection of the throat in connexion with it. But these are phenomena which characterise certain eruptive febrile diseases; and that erysipelas of the face is preceded and accompanied by such fever, it is unnecessary here to state. The accession of the febrile state previously to the appearance of the eruption,—the symptoms which characterise this state, more especially the peculiar affection of the sensorium, and the determinate course of the disease, are equally known; and, although I am aware that these remarks contain no novelty, still it appeared to me that, at a time when the term Erysipelas had attained such ubiquity of application, it might be advantageous to direct attention, in a more particular manner than has been recently done, to certain phenomena accompanying erysipelas of the face,—to advert to its pretensions to be considered as a distinct disease,—and to recall to notice its claims to be ranked amongst the order Exanthemata, from whence, since the days of WILLAN and BATEMAN, it has been improperly expelled.

But it will be said, does not, then, inflammation of the skin of the trunk and extremities occur as a consequence and symptom of the same febrile action as that of erysipelas of the face? To which I would reply, that it may; but that this is a frequent occurrence, seems negatived, by the fact of all systematic writers having selected this last disease for description; and personally I have not had an opportunity of witnessing a case where inflammation of the cutis of the trunk or extremities occurred in connexion with, and preceded by, the same febrile affection which characterises erysipelas of the face, or observing its determinate course. It is no contradiction to this statement to allow that occasionally in this disease the inflammation extends from the skin of the face to that of the neck and trunk: this is an exception to the general rule, and the primary affection is still of the face in the few cases where it does occur. That inflammation of the cutis, and to some extent, does sometimes take place in typhous fever, is only what this membrane shares in common with many others of the body in the course of this disease. That the skin may occasionally become inflamed to a greater or less degree and extent, in connexion with and symptomatic of other diseases, especially chronic visceral affections, is an accidental occurrence, and is neither accompanied by the fever of idiopathic erysipelas, nor observes its precise course. In those instances, again, where, from local injury, inflammation has attacked the integuments of the face, the febrile disturbance, when it occurs, is a consequence of the local affection. This is also the case with the vast majority of what are called cases of erysipelas, of the trunk and extremities: they are cases of inflammation arising from some local cause of irritation. For, whether the inflammation may have arisen from the irritation of leech-bites, that of a blistering plaster, from a wound or ulcer, or have supervened on integument distended by œdema, still all these, and many other instances where common inflammation of the skin to some extent takes place, have been considered and designated as cases of erysipelas. That no advantage, and considerable confusion both as regards the nature and treatment of disease, should arise from making use of the same term to mark a general febrile affection, and others of local origin, would seem pretty natural. If the inflammation of the skin, as such, is pretty much the same in either instance, and if our remedial means are also somewhat similar, still the principles which regulate the application of these means are considerably different in a diseased condition of local origin, and one which arises from a specific fever and is not to be cured but by guiding the patient through such fever.

But the term Erysipelas has not been limited in its application to affections of the cutis only, and of various origin: it has been applied to those of other tissues, differing very essentially in their nature, and treatment when inflamed, from the cutis. Inflammation of the cellular substance and of the fasciæ have been comprehended under this term also, and that merely from the circumstance of their being attended with more or less redness or inflammation of integument.—

But the difference which such cases presented from common erysipelas were too striking to be entirely overlooked, and it was probably to meet them that the addition of the word Phlegmonodes was resorted to. When we come to inquire, however, what pathological state is designated by the phrase "Erysipelas Phlegmonodes," we shall find that it also is applied to more morbid conditions than one.

Simple erysipelas, in all its extent of application, is understood by those who employ it, to indicate inflammation of the skin, connected, it is true, in their belief with something mysterious in its nature. To the skin the affection is limited; and it has been well observed by CALLISEN,* "Telam autem cellulosam non invadit erysipelas nisi cum phlegmone nuptum." Phlegmonous erysipelas, then, would seem to signify erysipelas complicated with phlegmon; and we find both writers and practitioners describing as cases of erysipelas phlegmonodes, those of erysipelas of the face complicated with abscess of the eyelids, or of inflammation of the integuments of the trunk or extremities with the addition of a circumscribed abscess at one point of its extent.

But these do not form the majority of cases ranked under the head of Erysipelas Phlegmonodes. The cellular substance is a tissue very liable to become the seat of inflammation. This may originate spontaneously, or at least without any very evident cause,—more commonly it is the result of wounds, fractures, and ulcers,—some severe and extensive cases have been produced by injuries of bursæ, particularly those of the patella and olecranon; in the vicinity of the bladder it is occasioned by a special cause—infiltration of urine; and it exists on the most extensive scale in many of those cases of disease presumed to be the effect of the inoculation of a poison from a dead human body. Although the phenomena attending inflammation of the cellular substance are sufficiently characteristic, they are liable to some modifications; the extent, violence, and rapidity of the process being much influenced by the causes which have produced it. In most instances it is accompanied by more or less redness or inflammation of integument, but not as an essential character of the disease. This may be so slight as not to attract attention: indeed, the puffiness and extreme tenderness of the part inflamed, together with a colourless integument, have been noticed as matters of surprise; or it may have been present in a degree to be denoted by such expressions as "erythema," or "erythematous blush." But usually the affection of the

* Systema Chirurgiæ Hodiernæ, vol. i. p. 220.

skin is considerable; and it is to cases of this description, particularly as they occur in the extremities, that the term Erysipelas Phlegmonodes is most frequently applied; the real disease, the inflammation of the cellular substance, being lost sight of in the more visible consequence or complication, the affection of the skin. It is not necessary that individual instances should be referred to in proof of this statement; for whoever takes the trouble to examine them in their details, will find that, in the great proportion of cases classed under the head of Erysipelas Phlegmonodes, and in many of those of Traumatic Erysipelas, the affection of the cutis is the least important part of the affair. We may merely cite one, and that for the purpose of contrasting the propriety of the name with the diseased condition described in the quotation.

A labourer* grazed the skin over his shin-bone, and three weeks afterwards, when it was supposed to be well, he applied for advice on account of an ulcer in this situation, about the size of a split pea, surrounded with inflammation. Three days subsequently, Dr. Butter saw the man. A dull and unequal redness, not unlike deeply stained mahogany, now extended itself around the small part of the left leg, from the inner ankle to the calf. The redness was peculiarly mottled, not unlike Erythema Papulatum, figured in Willan, without vesications. Agonising pain prevailed, particularly on the inflamed parts of the limb, and increased by the slightest pressure. An incision, five inches in length, was made into the parts down to the fascia. "The divided edges gaped widely, and looked like sliced bacon or brawn. The epidermis, rete mucosum, and cutis vera, were thicker, denser, and redder than natural. The cellular substance was distended, and considerably raised above the muscles, by a yellowish, gelatinous, and semi-fluid substance, intermixed here and there with dots of pus, and whitish shreds of slough."

To this pathological condition, (the fidelity of the description will be recognised, at least as applicable to inflammation of the cellular substance where its texture is loose and free from fat, as in the leg and fore-arm,) the name of Erysipelas Phlegmonodes is given. But we may ask, with what justness? The term Erysipelas has been made use of to denote inflammation of the skin simply; Phlegmon to designate a circumscribed inflammatory tumor, having a tendency to suppuration. But the diseased condition here spoken of, is, it is unnecessary to say, neither phlegmon nor erysipelas, still

* Case of Reeves, in Dr. Butter's work on Irritative Fever; Devonport, 1825, p. 101.

less is it a combination of both. The skin may share inconsiderably in the inflammation, and that too but secondarily; and the wide extent of disease renders the character of a circumscribed tumor inapplicable. The phenomena, also, are not those of phlegmonous inflammation; and even if a phlegmon is to be regarded as the type of inflammation of the cellular substance, then the same term is applied to very different morbid states, according as it is used in an adjective or substantive sense. Should it be said, on the other hand, that its tendency to spread gives it an erysipelatous character, then it must be answered, that the mere extent of inflammation can give it no more pretensions to be considered as erysipelatous (implying a difference in kind) in the case of the cellular tissue, than in those of the peritoneum, mucous membrane of the bronchiæ, &c. where it is equally ready to spread.

The propriety of applying the term Erysipelas to every case of common inflammation of the skin, merely because it occupies some space, has been already hinted at; and when we come to consider the extent and continuity of the subcutaneous cellular substance, the surprise would rather seem to be as in the case of the skin, that it should be limited, since we know that in tissues which resemble both in the circumstances just mentioned, limitation of inflammation is the exception, not the rule. Previous to the adoption of the treatment of inflammation of the cellular substance by free incision, it might have been urged that another circumstance in favour of its erysipelatous nature was its having, like erysipelas, a certain mysteriousness of character. In so far as the cellular substance is concerned, (whatever may be the case with the cutis,) this argument can no longer be used, unless, indeed, it be said that the utility of this treatment depends on the mysterious principle making its escape at the free aperture given for its exit.

As if sufficient ambiguity had not already existed from confounding under the same name, affections both of the skin and cellular substance, those of the fasciæ also have been added. The head and extremities, where these membranes are most developed, are constantly presenting examples of their being attacked with inflammation; and to the phenomena attending which, the appellation of Erysipelas has been given. We may take the head for an illustration; for we there find, two very distinct affections of the coverings of the cranium designated by this name. In the idiopathic erysipelas of the face, it frequently happens that the inflammation extends from the integument of this part to that of the cranium, the cutis alone being affected, and where, as the process successively attacks different parts of this tissue, the line of progress is marked by an irregular but well-defined elevated edge, and within which line the texture itself of the tissue seems, as it were, more developed, presenting, in connexion with that of the unaffected part, an appearance something like that of embossed work. To this affection the name of Erysipelas of the Head may with propriety be applied. But, on the other hand, it is of no less frequent occurrence, and most commonly as the result of injuries, that the tendinous aponeurosis covering the cranium, the sub-aponeurotic and the cellular substance, become the seat of inflammation, in which case that of the cutis by no means necessarily follows. The part is, indeed, puffy, œdematous, and tender to the touch; but the tissue of the cutis does not present the alterations in texture observed in the preceding case, but is simply elevated by the disease in the subjacent parts. To this aponeurotic and sub-aponeurotic inflammation the term Erysipelas is applied. Both these affections may, it is true, exist at one time; for as in the extremities the same cause sometimes produces inflammation of the skin and of the cellular substance simultaneously, so here also an injury may excite this process in the cutis and aponeurosis at the same time. The affections, however, are distinct, so much so that the last-mentioned part not unfrequently, as a consequence of inflammation, suppurates, and separates in shreds from almost the entire cranium, (to which kind of case the name of Erysipelas Phlegmonodes has been sometimes applied,) without the integument suffering; a circumstance owing, as has been remarked by M. Dupuytren, to the two parts having each a separate set of vessels. The treatment of these affections,—to take the local, for example,—is very different: in the one (that of the cutis) local treatment is of little value or importance, and our applications are regulated chiefly by a regard to the gratification of the patient's feelings; in the other (that of the aponeurosis, or parts subjacent,) local treatment —free incision—is of the first importance and necessity.

That great confusion should have arisen from this unreserved application of the term Erysipelas, and the utter neglect of precision as attached to its meaning, is a consequence sufficiently natural, and a circumstance equally certain. To adduce evidence on this point would be superfluous, but it may be allowed me to cite one proof in illustration of the disadvantage accruing from the application of the same name to different diseases, and that from an author whose attention had been called to the subject of diagnosis. Dr.

Duncan, jun. in his Essay on Diffuse Inflammation of the Cellular Texture,* expresses an opinion that many cases of phlegmonous erysipelas ought to be referred to this head; an opinion in which, without assenting to the propriety of the term "diffuse," or to the manner in which the author has generalised, we may readily concur. On coming to the causes of the disease, (or rather of the diseases which Dr. D. wishes to consider as diffuse inflammation of the cellular texture,) he alludes to contagion as an obscure cause, and instances what occurred in Mr. Newby's family as the only fact which could lead to the suspicion of its having such an origin. How to reconcile the circumstance of the occurrence of erysipelas phlegmonodes in this instance from contagion, and the circumstance of the erysipelas phlegmonodes of Mr. Copland Hutchison never arising from such a cause, seems to have perplexed the author. He allows that the circumstance of so many persons being affected with disease in the family of Mr. N. was remarkable, but concludes by expressing an opinion that he cannot consider them as sufficient proofs of contagion. Had Dr. Duncan considered that the erysipelas phlegmonodes of Dr. Nelson was one disease, and that the erysipelas phlegmonodes of Mr. Hutchison was another, he would have had less difficulty, and probably might have allowed that idiopathic erysipelas of the face, with abscess of the eyelids, may be derived from contagion; whilst he might, with equal truth, have asserted that inflammation of the cellular substance in the extremities is not.

It is from the same cause—the almost unlimited application of the term, whence has mainly arisen the vagueness of ideas with regard to disease, associated and almost synonymous with the very name of Erysipelas. And it is, therefore, not surprising, that Mr. Travers should thus express himself in his recent work:—"To the term Erysipelas I object, as undefined in its application, complicated with endless varieties, and a perplexing catalogue of different species, which seems to augment in the hands of every additional describer."†

* Transactions of the Medico-Chirurgical Society of Edinburgh, vol. i. 1824, p. 584.

† "On Constitutional Irritation," London, 1826, p. 534.—It is to be regretted that, in objecting to the employment of the term Erysipelas as applied to an affection of the skin, Mr. T. should, in another part of his work, have lent the sanction of his authority to its retention and employment in what appears to me an equally questionable sense. At p. 205, he divides inflammation of the cellular substance into "phlegmonous, erythematous, erysipelatous, and gangrenous." So likewise Dr. Farr states (Appendix, p. 545,) that "the lymphatics are liable to an erythema."—Query, what is erythema of a lymphatic?

In our treatment, as in our ideas of the nature of disease, of what indecision, vacillation, and anomaly, is not this name of Erysipelas the parent? Some conceive erysipelas to be of a bilious nature; others imagine it to be of an inflammatory nature; whilst both agree with a third, that it is of a most mysterious nature. One treats all cases of erysipelas by tartarised antimony, another by bleeding, and a third by bark. In idiopathic erysipelas of the face, why should not the same principles which regulate and modify our treatment of other exanthematous fevers, equally apply? On the other hand, why should we not call inflammation of the skin, occurring as it does as a primary affection, by its name, and treat it as such? Is there any more difficulty in conceiving that inflammation of the skin should produce a peculiar effect on the general system, than that inflammation of the peritoneum, mucous membrane of the bronchiæ, &c. should? Is there any better reason for treating a case of inflammation of the skin, or of the cellular substance, solely by attention to general means, than that it is called Erysipelas? Is it not this name alone also, and the mysterious ideas associated with it, which leads to the strange anomaly of seeing inflammation treated by such a stimulant as wine? Because inflammation of the skin or cellular substance, like inflammation of the mucous membrane of the bronchiæ, of the urethra, and of the eye, is less under the influence of general blood-letting than inflammations of serous membranes and other tissues, ought we therefore to treat it by stimulating the system at large?—But this communication has already extended to too great a length.

On reviewing, therefore, these observations, and for the reasons developed in the course of them, I would venture, in conclusion, to submit—

1. That the term Erysipelas should be restricted to that febrile affection of the system, accompanied with inflammation of the integuments of the face, to which it has most usually been applied; and that, until we have better evidence for so doing, the expressions Erysipelas and Erysipelatous should not be applied to affections of the skin in other parts of the body.

2. That the term Erysipelas Phlegmonodes should be abandoned, as unnecessary as well as inaccurate, and applied to dissimilar morbid conditions.

3. That Inflammation of the Cutis of the extremities and trunk, it matters not from what cause or however extensive, should be designated simply as such; to the exclusion of the terms Erysipelas and Erythema, as improper and unnecessary, the one insinuating a difference in kind, the other implying merely a difference in degree.

4. That Inflammation of the Cellular Substance should be described as such, and by name; and, as no necessity exists for the addition of the term "diffuse," so long as we possess the adjective "extensive," that the adoption of the former word should be abandoned.

Lastly. That Aponeurotic and Subaponeurotic Inflammation should likewise be withdrawn from its erysipelatous disguise, and appear in its own proper character and name.

*New Burlington-street; Dec. 20th,* 1826.

P.S.—Owing to the delay in publishing the above remarks, I have had an opportunity of perusing the paper of Mr. EARLE, in the Number of this Journal for January; and I am happy to find that, in so far as regards the impropriety of the application of the term Erysipelas Phlegmonodes to the disease of which he treats, our ideas coincide.

*Jan.* 4, 1827.

